# Metagenomic characterization of a novel non-ammonia-oxidizing Thaumarchaeota from hadal sediment

**DOI:** 10.1186/s40168-023-01728-2

**Published:** 2024-01-08

**Authors:** Ru-Yi Zhang, Yan-Ren Wang, Ru-Long Liu, Sung-Keun Rhee, Guo-Ping Zhao, Zhe-Xue Quan

**Affiliations:** 1 Fudan Microbiome Center, Ministry of Education Key Laboratory for Biodiversity Science and Ecological Engineering, National Observations and Research Station for Wetland Ecosystems of the Yangtze Estuary, Institute of Biodiversity Science and Institute of Eco-Chongming, School of Life Sciences, Fudan University, Shanghai, China; 2 Shanghai Engineering Research Center of Hadal Science and Technology, College of Marine Sciences, Shanghai Ocean University, Shanghai, China; 3 Department of Microbiology, Chungbuk National University, Cheongju, Republic of Korea

**Keywords:** Hadal sediment, Metagenome, Novel heterotrophic Thaumarchaeota, Intermediary role in the evolution

## Abstract

**Background:**

The hadal sediment, found at an ocean depth of more than 6000 m, is geographically isolated and under extremely high hydrostatic pressure, resulting in a unique ecosystem. Thaumarchaeota are ubiquitous marine microorganisms predominantly present in hadal environments. While there have been several studies on Thaumarchaeota there, most of them have primarily focused on ammonia-oxidizing archaea (AOA). However, systematic metagenomic research specifically targeting heterotrophic non-AOA Thaumarchaeota is lacking.

**Results:**

In this study, we explored the metagenomes of Challenger Deep hadal sediment, focusing on the Thaumarchaeota. Functional analysis of sequence reads revealed the potential contribution of Thaumarchaeota to recalcitrant dissolved organic matter degradation. Metagenome assembly binned one new group of hadal sediment-specific and ubiquitously distributed non-AOA Thaumarchaeota, named Group-3.unk. Pathway reconstruction of this new type of Thaumarchaeota also supports heterotrophic characteristics of Group-3.unk, along with ABC transporters for the uptake of amino acids and carbohydrates and catabolic utilization of these substrates. This new clade of Thaumarchaeota also contains aerobic oxidation of carbon monoxide-related genes. Complete glyoxylate cycle is a distinctive feature of this clade in supplying intermediates of anabolic pathways. The pan-genomic and metabolic analyses of metagenome-assembled genomes belonging to Group-3.unk Thaumarchaeota have highlighted distinctions, including the dihydroxy phthalate decarboxylase gene associated with the degradation of aromatic compounds and the absence of genes related to the synthesis of some types of vitamins compared to AOA. Notably, Group-3.unk shares a common feature with deep ocean AOA, characterized by their high hydrostatic pressure resistance, potentially associated with the presence of V-type ATP and di-myo-inositol phosphate syntheses-related genes. The enrichment of organic matter in hadal sediments might be attributed to the high recruitment of sequence reads of the Group-3.unk clade of heterotrophic Thaumarchaeota in the trench sediment. Evolutionary and genetic dynamic analyses suggest that Group-3 non-AOA consists of mesophilic Thaumarchaeota organisms. These results indicate a potential role in the transition from non-AOA to AOA Thaumarchaeota and from thermophilic to mesophilic Thaumarchaeota, shedding light on recent evolutionary pathways.

**Conclusions:**

One novel clade of heterotrophic non-AOA Thaumarchaeota was identified through metagenome analysis of sediments from Challenger Deep. Our study provides insight into the ecology and genomic characteristics of the new sub-group of heterotrophic non-AOA Thaumarchaeota, thereby extending the knowledge of the evolution of Thaumarchaeota.

Video Abstract

**Supplementary Information:**

The online version contains supplementary material available at 10.1186/s40168-023-01728-2.

## Introduction

Archaea of the phylum Thaumarchaeota are ubiquitously distributed on Earth, with the majority of them classified as lithotrophic ammonia-oxidizing archaea (AOA), having an important contribution to the global nitrogen cycle, particularly in the oceans [1–4]. However, this phylum also comprises members that lack the key enzymes for ammonia oxidation, which are classified as non-AOA Thaumarchaeota [3, 5]. In contrast to the reported AOA Thaumarchaeota, which are affiliated with the class Nitrososphaeria [6], heterotrophic Thaumarchaeota are deeply branched in the phylum [3, 7, 8]. Non-AOA Thaumarchaeota were first identified in terrestrial systems such as anoxic peat soils [9], subsurface aquifer sediment [10, 11], geothermal springs [12, 13], and acidic forest soil [5]. In the marine system, lineage psL12 belonging to Group-3.b of non-AOA Thaumarchaeota was identified, which was characterized by aerobic heterotrophic ability [7, 8].

Although trench environments possess environmental characteristics (e.g., temperature, salinity, and oxygen) similar to those of the abyssal oceans [14–16], the extreme hydrostatic pressure and hydrotopographical isolation of the trench result in the creation of hadal environments. Microbes in hadal environments have characteristics of a barophilic nature, a high rate of carbon turnover, and a distinct ecological distribution [14, 17–19]. Owing to the funnel effect, dissolved organic matter (DOM) is concentrated in hadal sediments, leading to a different microbial taxa composition characterized by high cell abundance and strong microbial carbon turnover, compared with hadal waters and abyssal plain sediments [19–21]. The main DOM components of the deep ocean are refractory dissolved organic carbon (RDOC) such as polycyclic aromatics and highly aromatic compounds [22, 23]. Through culture-independent amplicon analysis, Thaumarchaeota was determined to be predominant archaea not only in hadal water [21, 24] but also in the hadal surface sediment [20, 25–27]. Systematic metagenomic studies of hadal water revealed that while Thaumarchaeota was the predominant archaea, the AOA community composition varied with depth [28]. Compared with the AOA Thaumarchaeota in shallow water, those inhabiting the deep sea have developed novel mechanisms to cope with extreme conditions, for example, V-type ATP synthase genes adapted to high-pressure conditions [29], putative di-myo-inositol-phosphate synthase genes for the synthesis of osmolyte [30, 31], and more transporters of organic compounds for the utilization of sinking organic carbon [28, 32]. Recently, metagenomic analysis of the Yap and Mariana Trench waters revealed the dominance of AOA Thaumarchaeota among archaea [33, 34]. However, systematic and detailed genomic research on the entire Thaumarchaeota community in the hadal sediment is limited.

In this study, through analysis of the Challenger Deep sediment and public datasets, one new metagenome-assembled genome (MAG) of a non-AOA Thaumarchaeota group was assembled. Our global distribution investigation based on public datasets indicated that this novel Thaumarchaeota clade, named Group-3.unk, exhibits a preference for inhabiting hadal sediment but is also prevalent in marine, terrestrial, and extreme environments. Pan-genomic analyses further suggested factors that lead to the ecological niche adaption of this new heterotrophic Thaumarchaeota. Evolutionary and gene dynamic analyses were also performed, which can provide insight into the new diversification of Thaumarchaeota.

## Materials and methods

### Mariana Trench sediment sampling and metagenome sequencing

The 0- to 10-cm-depth sediment samples were collected from the Challenger Deep of Mariana Trench (11.4037°N, 142.3630°E, 10 853 m) during the MV Zhangjian cruise from December 2016 to January 2017. One sediment column was collected using the Hadal Lander [35] equipped with a box corer. Detailed sample preparation and metagenome library construction processes were performed as described in previous studies [25, 36]. Raw data were deposited in the National Center for Biotechnology Information (NCBI) database and can be accessed under project ID PRJNA692099 [36].

### Assembly, bin refinement, classification, and phylogenetic analysis

The datasets were processed in the following manner. Fastqc (https://www.bioinformatics.babraham.ac.uk/projects/fastqc/) was used to check the sequencing state and quality of the reads. Trimmomatic (v0.39) [37] was used to remove low-quality reads and sequencing adaptors with the parameter ILLUMINACLIP:2:30:10:2 LEADING:3 TRAILING:3 MINLEN:36. Only paired high-quality sequences were used for assembly.

Data from sediment collected at different depths were assembled separately using the assembly tool St. Petersburg genome assembler (SPAdes) (v3.13.0) [38] in meta mode, and nine scaffold files were obtained. Clean paired reads of the samples were mapped onto the corresponding scaffold files using Burrows-Wheeler Aligner [39] in MEM mode. Nine scaffold files were binned with MetaBAT2 [40] individually. Bins from the nine samples were further refined using DAS_Tools [41] and estimated using CheckM [42]. High-quality MAGs (completeness ≥ 70% and contamination ≤ 5%) from individual assemblies were pooled and duplicated using dRep (v2.6.2) [43] with default parameters, and the MAGs were then classified using using Genome Database Taxonomy toolkit (GTDB-Tk) (v1.3.0) [44]. Multiple sequence alignment of 122 archaeal marker genes selected by GTDB-Tk for genomic classification was used to reconstruct the phylogenomic tree with the Thaumarchaeota reference genome. The phylogenomic tree was inferred using IQ-TREE (v2.1.2) [45] in PROTGAMMALG and node support was calculated based on a bootstrap value of 1000. The tree was visualized using the online tool Interactive Tree Of Life (iTOL) [46]. The Average Nucleotide Identity (ANI) value was calculated using pyANI (v0.2.10) [47]. Considering the distinct environment sources and metabolic characteristics of non-AOA Thaumarchaeota lacking the ammonia monooxygenase gene (*amo*A) [7–9, 12, 13], their clustering groups were proposed by referring to previous phylogenomic and pan-genomic studies [6, 8].

### Metagenomic read-based community constitution and metabolic coverage analysis

Reads from each metagenomic sample were mapped to the dereplicated MAGs using CoverM (0.6.1) (https://github.com/wwood/CoverM) with the “contig” command and the following parameters: methods, trimmed_mean; min-read-percent-identity, 95; and min-read-aligned-percent, 75. The coverage of each contig was calculated, and the coverage of each MAG was normalized by length. The relative abundance of MAGs in each metagenomic sample was calculated as coverage divided by the total coverage of all genomes in the dereplicated MAGs dataset. The classification of the dereplicated MAG dataset was determined using GTDB-Tk with the classify_wf command. METABOLIC (v4.0) was used to quantify genome and transcript coverages in the microbiome, microbial metabolic handoffs and exchange, and reconstruction of functional networks [48].

### Annotation and pathway construction

PROkaryotic DYnamic programming Gene-finding ALgorithm (Prodigal) (v2.6.2) [49] was used to predict coding DNA sequences and translated protein sequences in meta mode. All genomes were annotated using a local version of the annotation tool KofamScan (v1.3.0) [50] based on the Kyoto Encyclopedia of Genes and Genomes (KEGG) annotation profile database with default parameters. Mapper results from KofamScan were submitted to KEGG [51] for pathway construction. Subsequently, InterProScan (v5.38–76.0) [52] and EggNOG-mapper (v2.1.5) [53] were used to complete the annotation. The genes from different annotation results with different software were manually curated in UniProt [54] through BlastP (*e* value, 10^−5^) against the UniProtKB reference proteome plus Swiss-Prot database, and the top hits were selected as curated results. Transporter annotations were performed in TransportDB 2.0 [55] and curated in the Transporter Classification Database [56] with default parameters.

### Pan-genomic, functional, and metabolic comparison analyses

Thaumarchaeota MAGs identified in this study and reference genomes were submitted to an analysis and visualization platform for omics data (anvi’o) (v7.0) [57] for pan-genomic, functional, and metabolic comparison analyses. Thaumarchaeota genomes from public databases and this study with more than 70% completeness and less than 5% contamination were selected for detailed genomic comparison analysis according to the microbial pan-genomics and metabolism workflow. Gene annotation was performed on *anvi-run-kegg-kofams* based on the KEGG profile database. Pan-genomic analysis was conducted using the script *anvi-pan-genome*. BlastP was used for amino acid sequence similarity calculation, and the Markov cluster algorithm [58] was used for gene cluster identification based on amino acid sequence similarity. For the high-level taxa of phylum Thaumarchaeota, soft parameters (minbit = 0.5 and mcl-inflation = 2) were used for distantly related genomes. The script *anvi-compute-functional-enrichment* was used for functional enrichment analysis. The result of comparative genome analysis was visualized by ggtree [59]. The protein sequence of CoxL, a subunit of aerobic carbon monoxide dehydrogenase (CODH) was confirmed using BlastP against the customized reference database and the results from function annotation, and the CoxL sequences with the active-site motifs [13, 60] were selected for further phylogenetic analysis.

### Global distribution of non-AOA Thaumarchaeota

The global distribution of non-AOA Thaumarchaeota was estimated using read recruitment as described in a previous study [61]. In total, 234 Sequence Read Archive (SRA) datasets from various types of samples were downloaded from the NCBI SRA database. To avoid the disturbance due to ribosomal RNA (rRNA) genes to the accuracy of abundance measurements, Bacterial ribosomal RNA predictor (Barrnap) (v0.9) (https://github.com/tseemann/barrnap/) was used to predict the rRNA genes of the analyzed genomes. The Bedtools (v2.27.1) [62] command *maskFastaFromBed* was used to mask rRNA sequences of genomes. Recruitment was performed using BLASTn, and the hits were filtered based on a length cutoff of 50 bp, an identity cutoff of 95%, and an *e*-value cutoff of 10^−5^. These cutoffs were used to identify approximately similar genomes at the species level [63]. Qualified hits were used to compute the read counts per kilobase of genome per gigabase (RPKG) of the metagenome, which reflects normalized abundance, allowing comparison across different genomes and metagenomes.

### Reconstruction of more Group-3.unk clade genomes

Raw reads were obtained from the SRA datasets, consistent with the analysis of the global distribution of Thaumarchaeota genomes. Based on the distribution and abundance of MT1_thaum1, which binned from the trench sediment, the top 14 candidate metagenomes were selected for MAG reconstruction, and filtered according to the RPKG values of MT1_thaum1. Individual de novo assembly and binning procedures were performed as described above. Notably, for SRR10168429, the MT1_thaum1-like MAG from the raw individual assembly exhibited low completeness. Consequently, the MT1_thaum1 genome was utilized as a reference and BLASTn was employed to recruit the SRR10168429 metagenome, which was subsequently used to repeat the de novo assembly process.

### Gene dynamics, optimal growth temperature (OGT) prediction, timing estimation, and organic metabolism potential

To better understand the evolutionary history of Thaumarchaeota and avoid deviations introduced by genome incompleteness, 81 genomes with completeness > 80% and contamination < 10% were subjected to further evolutionary analysis. A phylogenomic tree was constructed as described above. To gain insights into the evolution of Thaumarchaeota genomes, filtered proteomes were input into OrthoFinder (v2.5.2) [64] with default parameters except for “-M msa,” and orthologous gene families were obtained. Annotation and categorization of these gene families were conducted using EggNOG-mapper. Along with the matrix of these gene families in the genomes, we used the COUNT program [65] to infer the history of genetic events (presence, gain, and loss) using the gain–loss-duplication model and posterior probability with default parameters. To assess the evolutionary history of the Thaumarchaeota phylum, its common ancestors were identified, and the gain and loss events and the presence of orthogroup gene families were determined along with a birth-and-death model for each evolutionary node and branch in the COUNT program with Dollo parsimony [65]. OGT prediction of highly qualified bins was performed using the Tome tool [66]. Additional 45 archaeal genomes [67] were included with the high-quality genome sets in this section for evolutionary analysis and were employed to compute key occurrence times across the evolutionary history of the Thaumarchaeota phylum (Table S1). The methods used for constructing the phylogenomic tree were the same as described above. Subsequently, the timeline analysis of the phylogenomic tree was conducted by Reltime [68, 69], with DPANN archaea as the outgroup, and six archaeal scenarios were selected as the calibration. The estimation results of AOA Thaumarchaeota were consistent with the findings of the previous study [67] (Table S2), establishing the reliability of this timing estimation analysis. Organic metabolism potential was determined by the proportion of organic metabolism-related archaeal Clusters of Orthologous Genes (arCOG) accounting for all annotated genes.

## Results and discussion

### Dominant Thaumarchaeota involved in DOM degradation

**Fig. 1:**
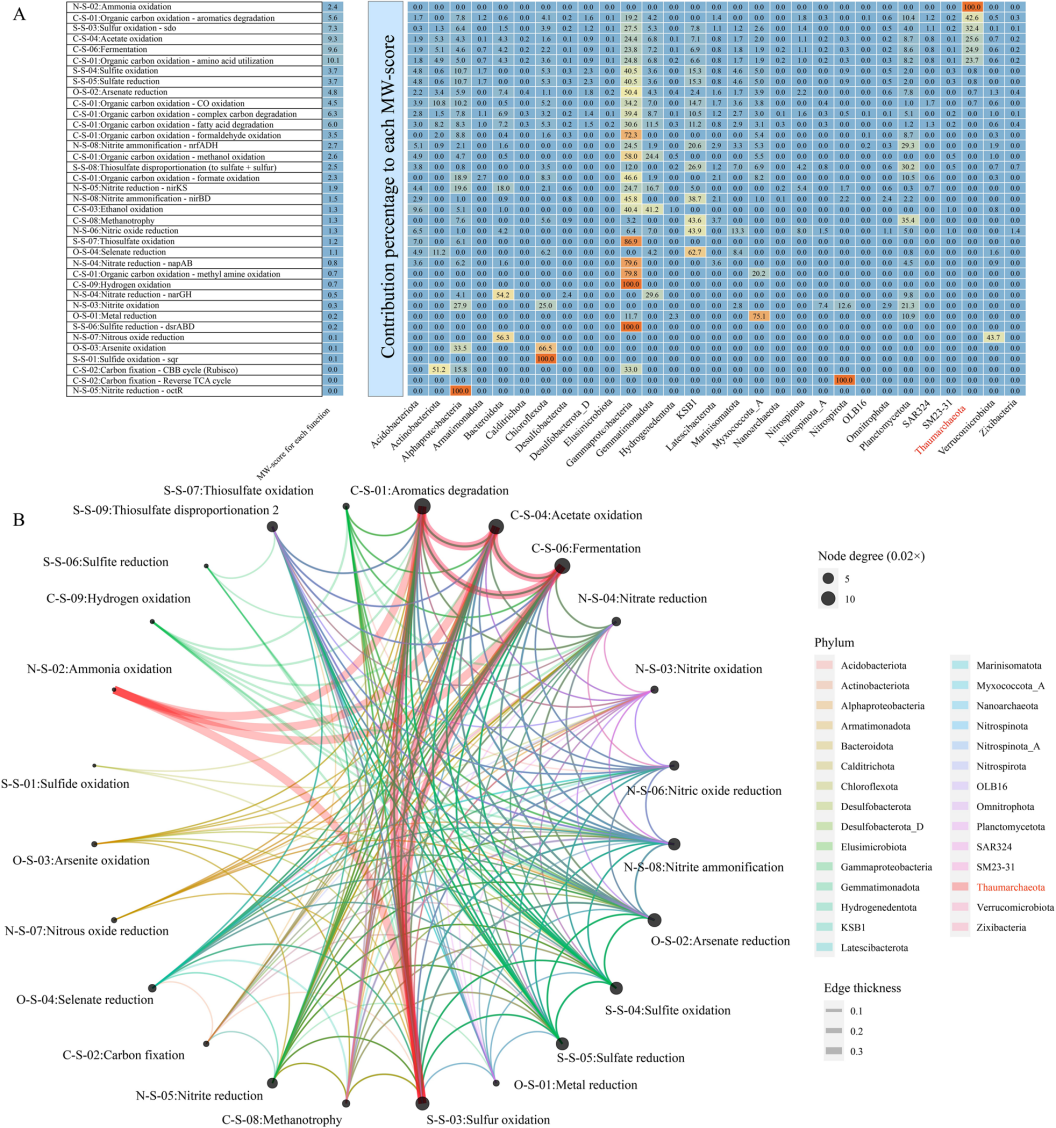
Metagenomic read-based metabolic analysis. **A** The term metabolic weight (MW)-score for each function was calculated using the software of METABOLIC [48], which indicates the functional weights within the whole community. The term contribution percentage to each MW-score reflects the contribution of each phyla to the function within the whole community. Thaumarchaeota shows a high percentage contribution to the MW scores of ammonia oxidation, aromatic degradation, sulfide oxidation, acetate oxidation, and amino acid utilization. The details of main enzymes of these pathways are presented in Table S3. **B** Node degree indicates the number of connections to each node. The thickness of a given edge is based on the average gene coverage values of two biogeochemical cycling steps

The microbial community in all 0- to 10-cm-depth sediment samples of the Challenger Deep in Mariana Trench displayed a remarkable dominance of Proteobacteria in bacteria and Thaumarchaeota in archaea (Fig. S1). Metagenomics analysis revealed that the microbial community of the Mariana Trench sediment had a high metabolic weight (MW)-score (representing the functional gene coverage) in DOM degradation-related pathways (Fig. 1A). The top-ranked DOM degradation pathways were amino acid oxidation (MW-score = 10.1), fermentation (MW-score = 9.6), and acetate oxidation (MW-score = 9.3), in which Thaumarchaeota contributed 23.7%, 24.9%, and 25.6% to the MW-scores, respectively. Furthermore, the microbial community largely contributed to the gene coverage related to aromatic substance degradation (MW-score = 5.6) with the main contribution of Thaumarchaeota (42.6% of the MW-score). The details of the main enzymes for these pathways are presented in Table S3.

Owing to their low biological reactivity [70], aromatic substances constitute a significant proportion of deep-ocean DOM and are considered key components of RDOC [22]. The massive gene contributions of Thaumarchaeota to RDOC degradation (Table S3), including flavin prenyltransferase (UbiX) [71], suggest that Thaumarchaeota may have the potential to metabolize RDOC and its intermediates under limited DOM conditions. The functional network showed a high-frequency connection (top one node degree) between the degradation of organic matter (such as aromatics degradation, acetate oxidation, and fermentation) and the oxidation of inorganic matter (such as ammonia oxidation and sulfide oxidation) particularly in Thaumarchaeota (Fig. 1B). This suggests an ecological connection between the microbial degradation of organic matter and the oxidation of inorganic matter at the surface of the trench sediment and facilitated by Thaumarchaeota.

A previous metagenomic and transcriptomic study identified a high relative abundance of sequence reads of genes and transcripts involved in the degradation of acetate and aromatic substances by microbial communities in the Challenger Deep sediment [34]. However, the specific contributors to the highly expressed corresponding metabolic genes remain unclear. Therefore, through reanalysis of these metatranscriptomic data from Ying et al. [34], we found that Thaumarchaeota contributed 23.3% of the transcripts to aromatic substance degradation, 11.0% to acetate oxidation, and 10.4% to fermentation (Table S4), further suggesting its significant role in DOM degradation. Therefore, in addition to ammonia oxidation, Thaumarchaeota (including AOA, the dominant Thaumarchaeota as described below) also occupies heterotrophic niches for DOM degradation in the sediments of Challenger Deep.

To better explore the cellular functional potential, we recovered 60 high-quality MAGs (Table S5) from dereplicated MAGs retrieved through de novo assembling strategies. The assembled MAGs contained two Thaumarchaeotal bins obtained from multiple assembly results of 0–2-cm-depth (bin MT1_thaum1) and 7–8-cm-depth (bin MT7_thaum2) sediments. Based on the contig sequence coverage in the metagenomes of the MT1 sample, one cluster of contigs from the Thaumarchaeota bin MT7_thaum2 was identified as the most abundant population (Fig. S2), which further confirmed the dominance of Thaumarchaeota in the Challenger Deep sediment among archaea.

### Phylogenetic analysis of Thaumarchaeota

**Fig. 2:**
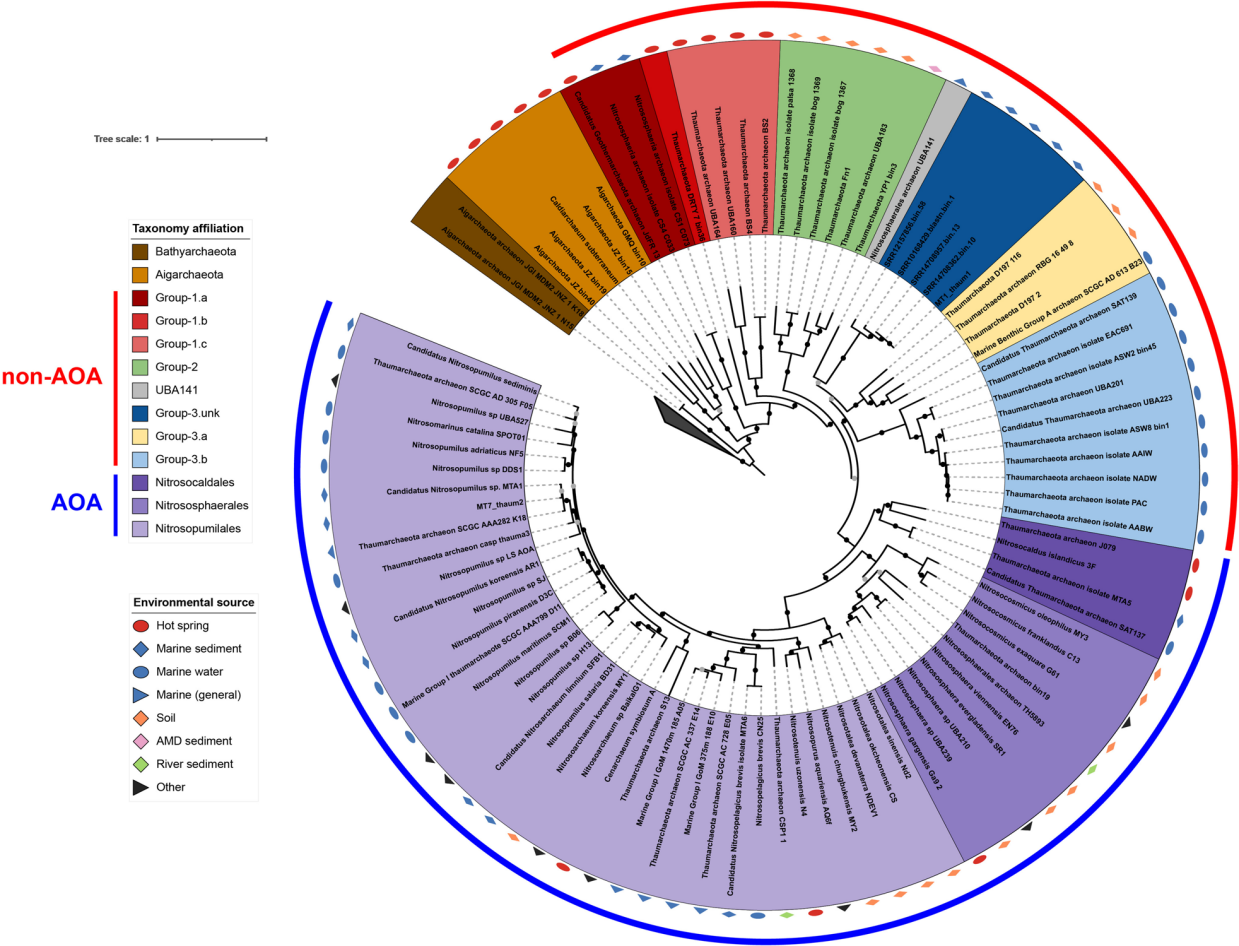
Phylogenomic tree of the assembled Thaumarchaeotal metagenome-assembled genomes (MAGs). The tree includes two Bathyarchaeota, five Aigarchaeota, and 85 Thaumarchaeota (51 ammonia-oxidizing archaea [AOA] and 34 non-AOA) genomes, with two MAGs assembled in this study. Phylogenomic analysis is inferred based on the concatenated alignment of 122 archaeal marker proteins as applied in IQ-TREE with 1000 bootstrap re-samplings. Legends of taxonomy affiliation and environmental sources of genomes are shown. Nodes with bootstrap values in the ranges of (50-70) and {70,100} are shown as grey and black dots, respectively. The collapsed clade indicates outgroup

MT7_thaum2 has high similarity (ANI value, 0.97) to Candidatus_Nitrosopumilus_sp_MTA1, (Fig. S3) which was binned from Mariana Trench water samples obtained at a depth of 8000 m in a previous study [28]. Phylogenetic analysis was conducted using two Bathyarchaeota, 5 Aigarchaeota, and 85 Thaumarchaeota (Table S6) to provide a well-supported Thaumarchaeota phylogenomic tree (Fig. 2). MT1_thaum1 and other four related MAGs reconstructed from public metagenomic datasets (ANI value > 0.82 with intra comparison) were associated with Group-3 but do not belong to Group-3.a/b (ANI value < 0.73) (Fig. S3); they also exhibit relatively long phylogenetic distances to UBA141 [72]. Based on this pattern, we propose this group as a new clade of heterotrophic non-AOA Thaumarchaeota, which was named Group-3.unk.

Extensive genomic and transcriptomic studies on heterotrophic Thaumarchaeota have not been conducted in the hadal waters or sediment to date. Our results indicate that the emergence of ancestral Thaumarchaeota was followed by diversification into three major non-AOA lineages with distinctive habitats: Group-1, hot springs; Group-2, anoxic soil; and Group-3.a, soil environment; and Group-3.b (psL 12), marine waters. However, except for the trench sediment analyzed in this study, the complete ecological niche of Group-3.unk is unknown.

### Ubiquitous distribution of Group-3.unk Thaumarchaeota

**Fig. 3:**
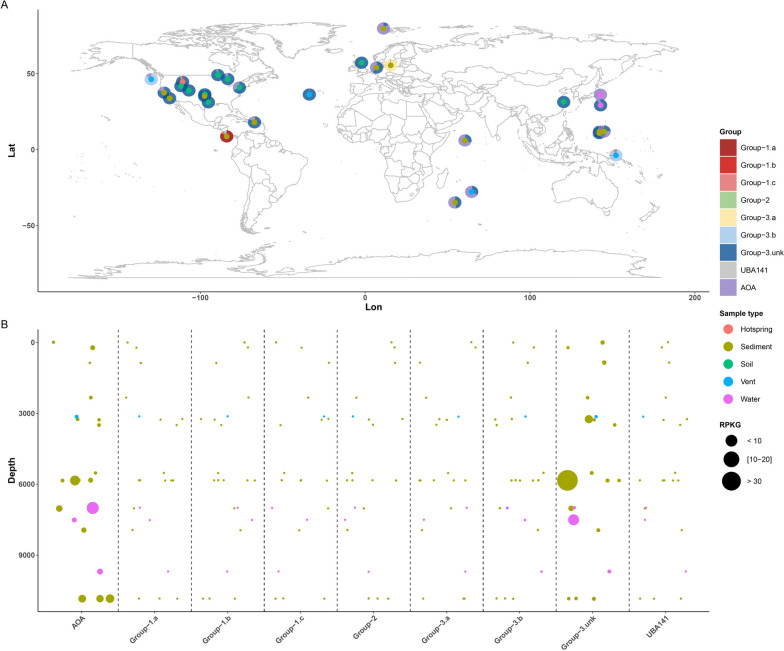
Global distribution of different group Thaumarchaeota. **A** Survey of Thaumarchaeota of 234 Sequence Read Archive (SRA) datasets. Group-3.unk top-ranked samples are shown on the map. Overlapped points are removed and only representative samples are shown. Group-3.unk non-AOA are ubiquitously distributed in series-type samples. **B** Relationship of the reads, expressed as recruited per kilobase of genome per gigabase (RPKG), for different group Thaumarchaeota and the depth of marine sample sites

The global distribution assessment of Group-3.unk demonstrated that this group inhabits diverse environments, including terrestrial, marine, and even extreme environments (e.g., hadal water and sediment, hot springs, and hydrothermal vents). Although Group-3.unk Thaumarchaeota accounted for the entire Thaumarchaeota phylum in some soil and sediment samples (Fig. 3A), Group-3.unk showed a relatively low absolutive quantity of genomes according to low RPKG value (< 0.1) in most samples (Table S7), which can explain why these genomes have not been binned in previous studies. However, in samples from a depth exceeding 6000 m, some of RPKG values for Group-3.unk reached up to > 30 (Fig. 3B, Table S8), which indicates a specific niche preference of this group to hadal environments.

Trench environments are considered depocenters for organic matter and are less oligotrophic than the upper layers of the ocean; production in these environments is considered to be driven by the sinking organic matter [14, 73], in which the RDOC increases with increasing depth [22]. To adapt to the unique conditions of the trench niche characterized by enriched RDOC and high hydrostatic pressure, Group-3.unk may have developed piezophilic adaptations and extended chemoorganoheterotrophic capacities, similar to those of the recently reported Group-3.b [7, 8], a sister group of Group-3.unk.

### Potential traits of the Group-3.unk clade

**Fig. 4:**
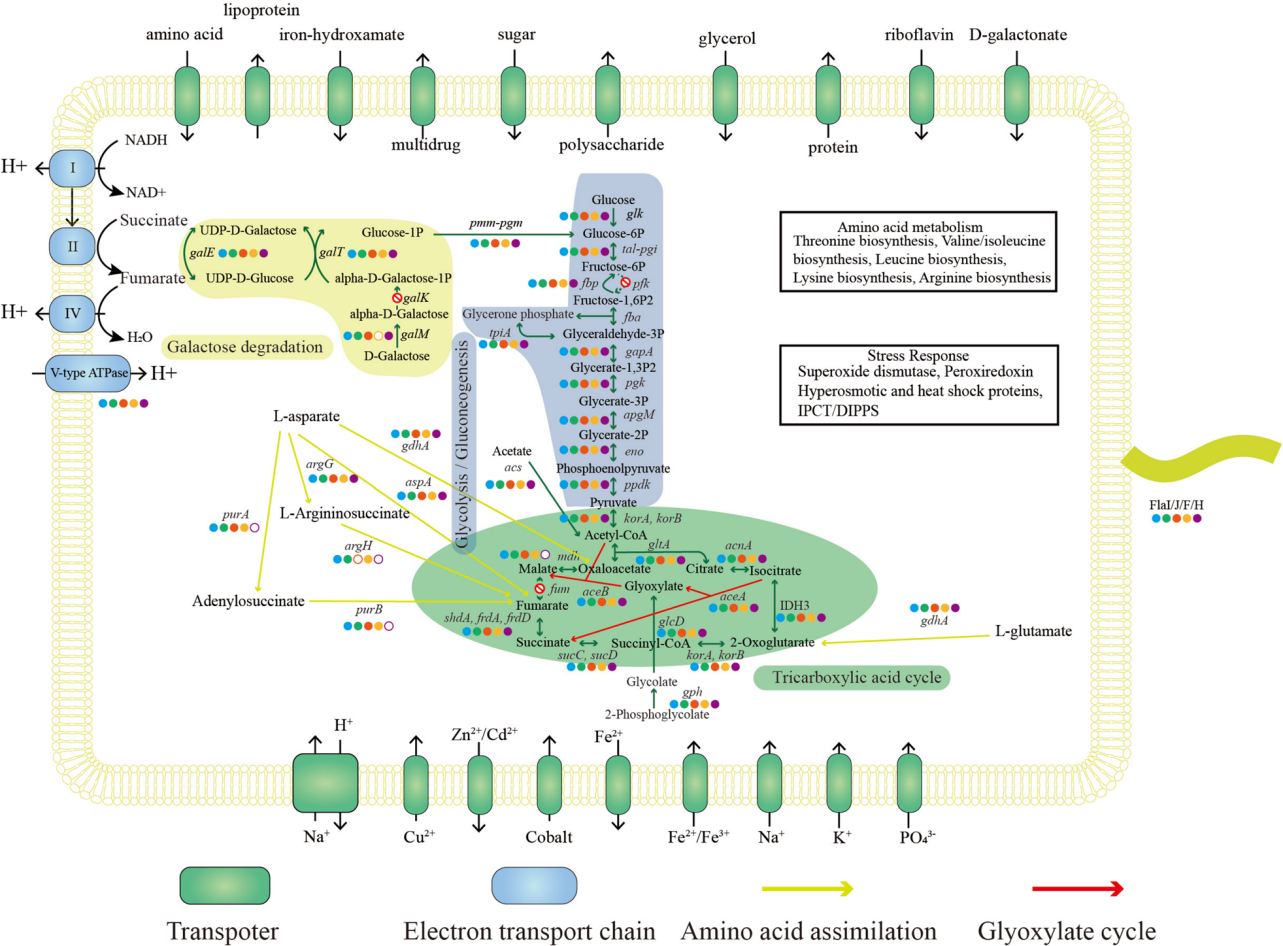
Schematic pathway reconstruction of the five metagenome-assembled genomes in Group-3.unk of non-ammonia-oxidizing archaea (non-AOA) based on multiple annotations. The five colored dots indicate MAGs from this study, in the order of MT1_thaum1, SRR10168429_blastn_bin_1, SRR12157856_bin_58, SRR14708362_bin_10, and SRR14708957_bin_13. Glycolysis/gluconeogenesis, complete tricarboxylic acid (TCA) cycle and galactose degradations are shown with different backgrounds. Amino acid metabolism and stress response information are shown in the black box

The reconstructed metabolic pathway demonstrated that non-AOA Thaumarchaeota Group-3.unk is capable of heterotrophy. The Group-3.unk genomes possess genes encoding transporters that transport extracellular amino acids and carbohydrates into the cytoplasm for utilization via central carbon metabolism. The annotated gene of FlaI/J/F/H, which is the key gene involved in the biogenesis of archaeal flagellum biogenesis, has the potential for motility. Replenishment for the intermediate of tricarboxylic acid (TCA) cycle by transported extracellular amino acids, storage of carbon sources via gluconeogenesis, and bypassing the emission of two molecules of carbon dioxide within the TCA cycle to facilitate carbon anaplerotic reactions by glyoxylate cycles provide flexibility for Group-3.unk in utilizing the carbon source in extreme environments (Fig. 4).

#### Transporters

Multiple transporters, which could uptake organic substrates such as amino acids and dipeptides/oligopeptides were annotated (Fig. 4). All five Group-3.unk bins possessed transporters for glutamine, glutamate, or asparagine and these substrates can be converted to fumarate and 2-oxo-glutarate, which are the intermediates of the TCA cycle (Fig. 4). In addition, all bins also possessed ATP-binding cassette (ABC) superfamily transporters that may uptake sugar, galactonate, and glycerol. Detailed annotation information for the transporters of the five Group-3.unk bins is provided in Table S9. Putative genes encoding ABC transporters for simple oligo- and monosaccharides, amino acids, and glycerol were previously reported to be present in non-AOA heterotrophic marine Thaumarchaea [8] and autotrophic AOA Thaumarchaeota [74] despite of lack of solid verification of mixotrophic or heterotrophic natures of AOA [75]. The expression of ABC transporters, especially amino acid transporters, is reported to increase with ocean depth [76]. Notably, more transporter genes were identified in the AOA Thaumarchaeota members in hadal water than in those residing in the upper layers of the oceans [28]. The high proportion of organic arCOGs in Group-3.unk than AOA also supports this group has a great potential for organic metabolism (Fig. S4). This implies that the transporters for amino acid and hydrocarbons may be helpful for the adaptation of microbes of clade Group-3.unk to hadal environments.

The Challenger Deep in the Mariana Trench, as the deepest site on Earth, has limited DOM primarily comprising RDOC [22]. The adaptation to this extreme environment may have favored the presence of a wide range of transporters. Iron ion transporter and vacuolar iron transporter (VIT) homologs were also observed. VITs are known to play significant roles in the processes of storing iron and regulating iron homeostasis to avoid excessive iron accumulation that could cause cytotoxicity [77]. The uptake of iron ions from the extracellular environment and its accumulation may be helpful for cellular activities as an enzyme cofactor. The Group-3.unk genomes possess genes of encoding polysaccharide exporters (Table S9) and some polysaccharide-synthesis-related genes were also annotated (Table S10), indicating they may have potential for exopolysaccharide production, similar to *Nitrososphaera viennensis* [78] and *Candidatus* Nitrosocosmicus agrestis [79].

#### Central carbon metabolism

Group-3.unk bins possess a nearly complete glycolysis pathway except for phosphofructokinase, the lack of which could be caused by the assembly gaps. Using the complete gluconeogenesis pathway, Group-3.unk can convert malate to glucose 6-phosphate. In addition, all bins possess glyoxylate cycles for carbon anaplerotic reactions, which further highlights the metabolic versatility of Group-3.unk among Thaumarchaeota groups. Most AOA Thaumarchaeota fix carbon through the 3-hydroxypropionate/4-hydroxybutyrate pathway [80], while the majority of non-AOA members putatively utilize the ribulose-1,5-bisphosphate carboxylase [7, 8] or Wood–Ljungdahl pathway for carbon fixation [11]; however, key genes of all of these carbon fixation pathways are absent in Group-3.unk only with incomplete reversible TCA cycle. Annotations of the genes of central carbon metabolisms for all five bins in Group-3.unk are presented in Table S10.

#### Energy metabolism

Group-3.unk hosts a respiratory electron transfer chain that includes a gene cluster encoding part of cytochrome c oxidase (Table S10), indicating the use of oxygen as an electron acceptor. Most MAGs of Group-3.unk contain complete V-type ATP synthase gene clusters (Table S10) adapted to high-pressure conditions same as deep ocean AOA [29]. Moreover, the presence of large, medium, and small subunits of the aerobic carbon monoxide dehydrogenase (CODH) complex (CoxLMS) suggests that Group-3.unk may utilize CO under aerobic conditions [13, 81]. Phylogenetic analysis showed that CoxL of Group-3.unk can be classified as Form II CODH [82] with the functional motif (PYRGAGR) (Fig. S5), with a maximum similarity of 46% to that of Aigarchaeota JZ_bin19 [13]. The CO oxidation function of Form II CODH was experimentally confirmed in aerobic hyperthermophilic archaeon *Aeropyrum pernix* TB5 [83]. Since some MRC (Marine *Roseobacter* Clade) strains only with form II coxL are known to be unable to oxidize CO [84], further study is required to confirm that Form II CODH is involved in CO oxidation function in non-AOA Thaumarchaeota.

The production of CO by submarine hydrothermal activities [85, 86] and by widespread marine bacteria [85, 87] were reported. Although there is no report of CO production in the trench ecosystem up to now, a high abundance of CODH genes in the metagenomes of different deep seas were reported [85, 87]. Therefore, CO may be important for the survival of Group-3.unk in the hadal marine sediment.

### Functional differentiation revealed by comparative genomic analysis

**Fig. 5:**
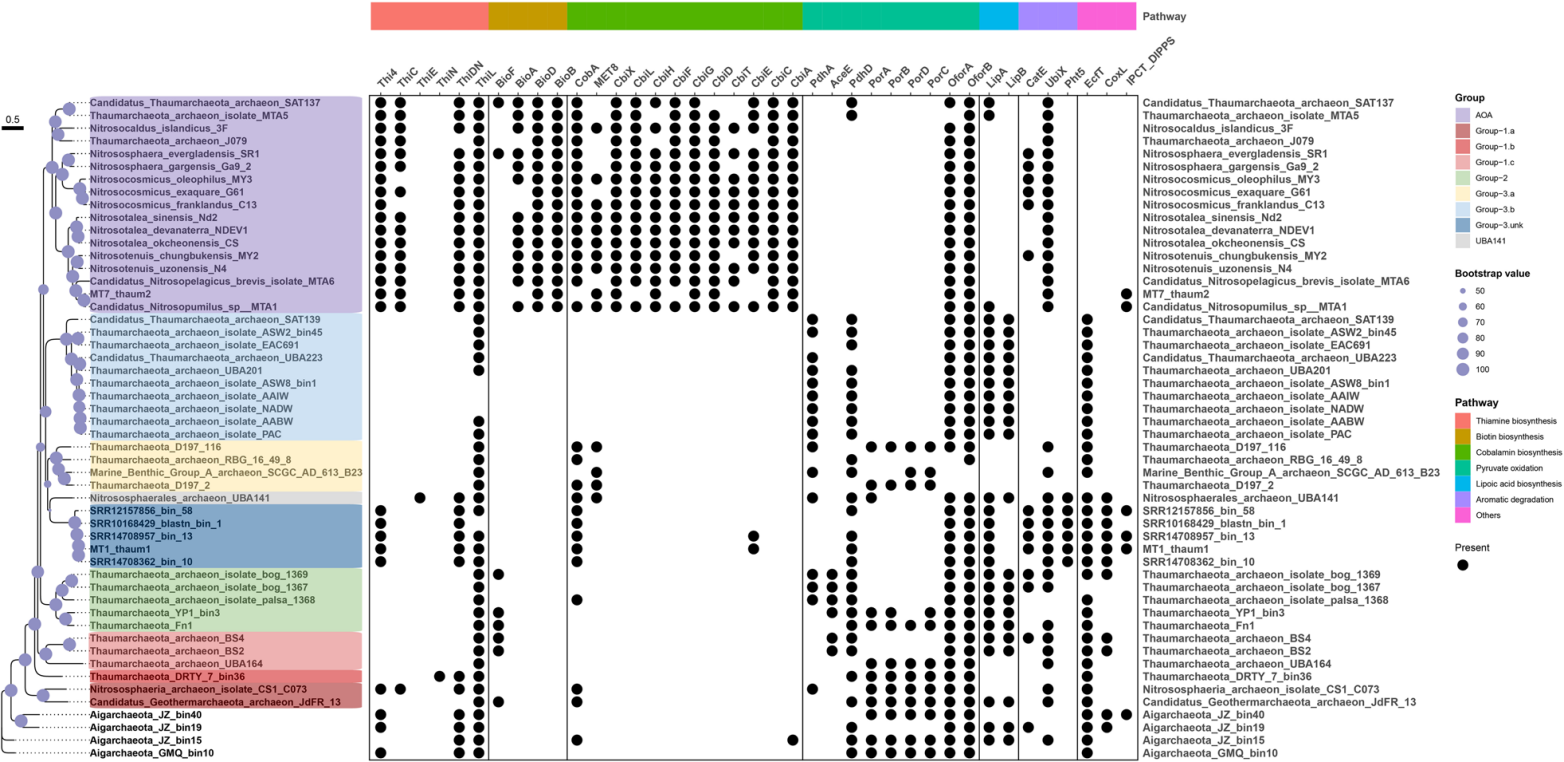
Illustration of comparative pan-genomic analysis results between groups of non-ammonia-oxidizing archaea (non-AOA) and AOA. MT1_thaum1 and MT7_thaum2 are metagenome-assembled genomes (MAGs) binned from Mariana Trench sediment samples. SRR12157856_bin_58, SRR10168429_blastn_bin_1, SRR14708957_bin_13, and SRR14708362_bin_10 are reassembled from public Sequence Read Archive (SRA) datasets. All Group-3.unk binned from this study are non-AOA, whereas MT7_thaum2 is an AOA group. Genes involved in thiamine biosynthesis, biotin biosynthesis, cobalamin biosynthesis, pyruvate oxidation, lipoic acid biosynthesis, and aromatic degradation are shown. PdhAD, pyruvate dehydrogenase E1 component subunit alpha/dihydrolipoyl dehydrogenase; AceE, pyruvate dehydrogenase E2 component; PorABCD, pyruvate ferredoxin oxidoreductase alpha/beta/gamma/delta; OforAB, 2-oxoglutarate/2-oxoacid ferredoxin oxidoreductase subunit alpha/beta; LipAB, lipoyl synthase AB; CatE, catechol 2,3-dioxygenase; UbiX, flavin prenyltransferase; Pht5, 4,5-dihydroxyphthalate decarboxylase; EcfT energy-coupling factor transport system permease protein; CoxL, aerobic carbon monoxide dehydrogenase large subunit; IPCT/DIPPS, inositol-1-phosphate cytidylyltransferase/di-myo-inositol-1,3′-phosphate-1′-phosphate synthase

As indicated by the distribution analysis and pathway reconstruction described above, Group-3.unk may have the ability to adapt to hadal environments. Indeed, pan-genomic and metabolic analyses demonstrated both the distinct characteristics of Group-3.unk Thaumarchaeota and common features with other Thaumarchaeota groups (Fig. 5).

Conspicuous differences were observed in vitamin synthesis between non-AOA and AOA Thaumarchaeota. VB_1_ (thiamine), VB_7_ (biotin), and VB_12_ (cobalamin) biosynthesis modules were found to be enriched only in the AOA group, as reported in previous studies [6, 88]. However, these vitamin synthesis-related genes were absent in all our analyzed non-AOA groups, including Group-3.unk; nevertheless, the non-AOA Thaumarchaeota harbored genes encoding transporters for the uptake of extracellular vitamins. The thiamine transport system present in non-AOA can offset a VB1 synthesis defect. In addition, almost all non-AOA Thaumarchaeota possessed ABC transporters (e.g., EcfT), which are responsible for the uptake of micronutrients such as vitamins from the environment [89–91].

Microorganisms adopt oxygen-sensitive pyruvate ferredoxin oxidoreductase (POR) [92, 93] and aerobic pyruvate dehydrogenase (PDH) reactions for the conversion of pyruvate to acetyl-CoA. In this study, subunits of 2-oxoacid ferredoxin oxidoreductase (OforAB) [94, 95] were identified in most MAGs of AOA and non-AOA Thaumarchaeota which may substitute PDH [96] (Fig. 5). MAGs of AOA Thaumarchaeota almost completely lost PorABCD and PDH complex (PdhD and AceE). Group-3.b and Group-3.unk members also do not contain POR, consistent with the results of previous studies on the loss of genes encoding POR in oxic non-AOA members [6, 88]. Genes related to lipoic acid synthase (LipAB), which is an important coenzyme for PDH and aerobic metabolism [97], are present along with the gene encoding PdhA in most MAGs (Fig. 5). Some of non-AOA Thaumarchaeota (YP1_bin3 and Fn1 in Group-2) contain both POR and OFOR (Fig. 5), and their obligate anaerobic respiration may be due to the lack of a cytochrome bd/c-type terminal oxidase [9]. It was predicted that loss of genes related to anaerobic energy production occurred during the evolution from non-AOA to AOA Thaumarchaeota, coinciding with the Great Oxygenation Event approximately 2300 million years ago [88]. Hence, the phenomenon described in this study may indicate a progressive loss of the capability of anaerobic growth in aerobic non-AOA Thaumarchaeota through transitioning from anaerobic (or facultative) non-AOA to aerobic non-AOA and finally becoming aerobic AOA.

The pan-genomic analysis revealed that non-AOA and AOA MAGs binned from the Challenger Deep sediment uniquely possess the osmoregulation-related gene encoding inositol-1-phosphate cytidylyltransferase/di-myo-inositol-1,3′-phosphate-1′-phosphate synthase (IPCT/DIPPS) (Fig. 5), which participates in the biosynthesis of di-myo-inositol phosphate, a key osmoprotectant, previously found in many hyperthermophilic archaea and bacteria [30, 31]. It was reported that marine group I deep-sea AOA binned from deep-sea water uniquely harbored the IPCT/DIPPS gene to deal with the hydrostatic pressure [32]. A recent study in Mariana Trench identified the expression of the IPCT/DIPPS gene using reverse transcription-quantitative PCR, and only detected the expression of IPCT/DIPPS in samples from the deep sea (> 4000 m), further emphasizing the requirement of IPCT/DIPPS for withstanding high hydrostatic pressure [28]. Intriguingly, through comparative genomic analysis (Fig. 5, Table S11), the IPCT/DIPPS gene was detected only in Group-3.unk and AOA from hadal environments, and was absent in all other Thaumarchaeota members binned from other environments. This indicates that IPCT/DIPPS could aid AOA and non-AOA Thaumarchaeota to adapt to extreme hydrostatic pressure.

In addition, Group-3.unk differentially possesses dihydroxyphthalate decarboxylase (Pht5) (Fig. 5), which plays a role in the degradation of polycyclic aromatic hydrocarbon by converting 4-hydroxyphthalate into 3-hydroxybenzoate or 4,5-dihydroxyphthalate into 3,4-dihydroxybenzoate [98]. Furthermore, all MAGs in Group-3.unk contain gene, encoding catechol 2,3-dioxygenase (CatE) which converts catechol to 2-hydroxymuconate-6-semialdehyde by opening the ring of benzol. This further supports the aromatic RDOC degrading trait of Group-3.unk in the hadal sediment.

### Gene dynamics from thermophilic to moderate non-AOA Thaumarchaeota lineages

**Fig. 6:**
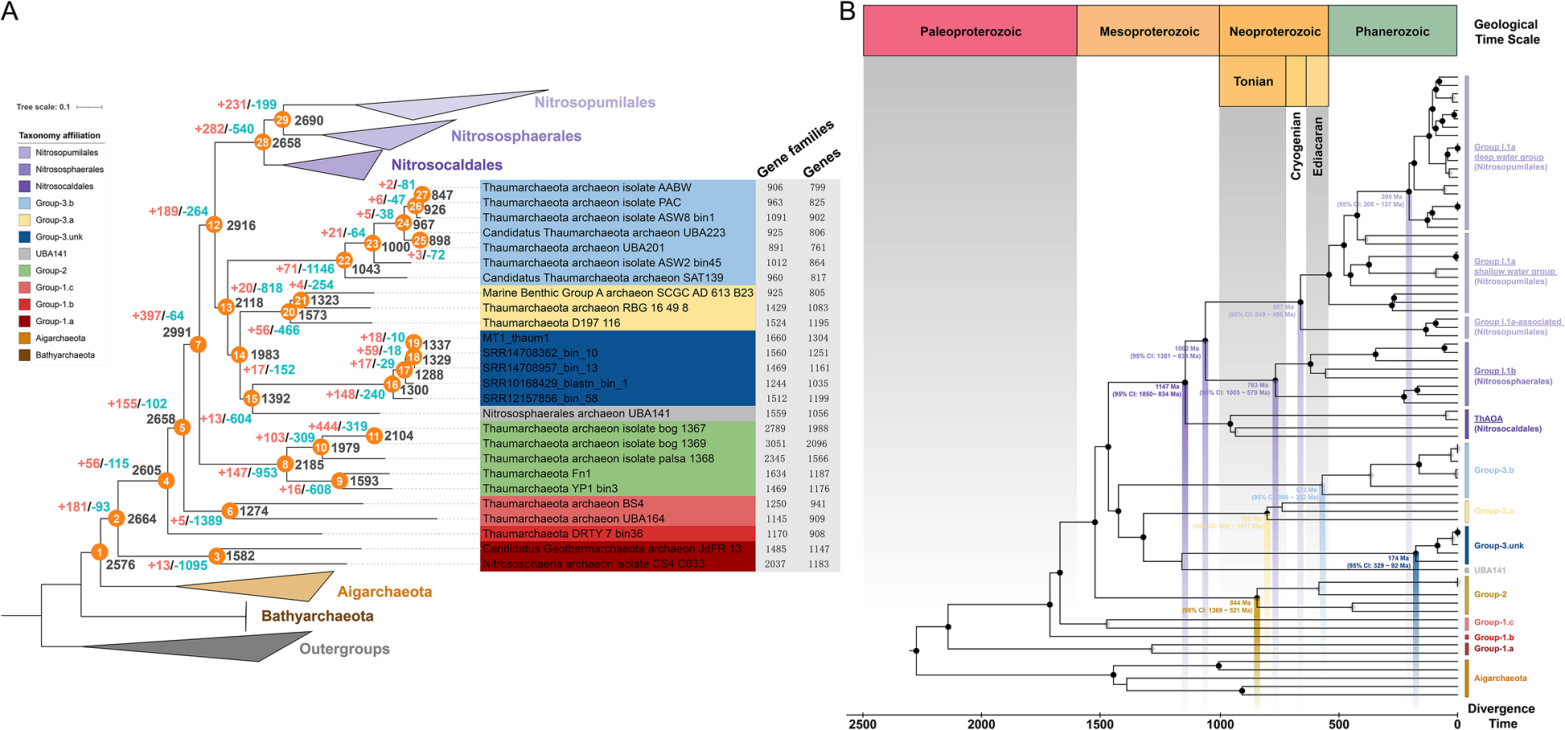
Evolutionary analysis of Thaumarchaeota. **A** Dynamic evolution of orthogroup gene families in Thaumarchaeota. Ancestral genome content reconstruction applied using COUNT. A number of genes are present and gene gain and loss events in the predicted ancestral genomes are marked at each lineage of the tree. Numbers at the right of the nodes represent the predicted present genes. “ + ” represents gene gain events, and “–” represents gene loss events. The topology tree was constructed based on the concatenated alignment of 122 archaeal marker genes from 81 high-quality genomes (completeness > 80%). Nitrosocaldales, Nitrosophaerales, and Nitrosopumilales in ammonia-oxidizing archaea (AOA) and Aigarchaeota all collapsed. **B** Timing estimation analysis between key nodes of Thaumarchaeota and the geological time scales throughout the Earth’s history. The tree added 21 Euryarchaeota, 16 Crenarchaeota, one Korarchaeota, two Bathyarchaeota, and two DPANN archaea (as outgroup). The added genomes of Thaumarchaeota are the same as in Fig. 6A. Complete tree is shown in Fig. S8. Nodes with bootstrap and data coverages (defined as the proportion of sites where there is at least one taxon in each descendent lineage that has available data to perform timing estimation) in the ranges of (70-100) and (50-80) are shown as gray dots; bootstrap and data coverage in the ranges of (70-100) and (80-100) are shown as black dots. The time unit at the bottom is millions of years (Ma), which corresponds to the geological time periods in the top panel

Gene gain and loss events, to some extent, could be used to infer the evolutionary process of prokaryote environmental adaptation [99]. Therefore, we analyzed the dynamics and ancestral reconstruction of Thaumarchaeotal and Aigarchaeotal genomes based on gene families of orthologous proteins from the 81 selected genomes. Aigarchaeota and Thaumarchaeota formed a monophyletic group, and their common ancestor showed 2576 orthologous gene families (node 1 in Fig. 6A and Table S12), not only including genes encoding enzymes involved in denitrification (COG0243 and COG4263) and the key anaerobic enzyme of pyruvate formation (e.g., ferredoxin oxidoreductase, COG0674, COG1013, and COG1144), but also the terminal oxidases, including heme-copper cytochrome c oxidase (COG1622, COG1845, and arCOG08921) and cytochrome bd ubiquinol oxidase (COG1271). It means the common ancestor of Aigarchaeota and Thaumarchaeota may potentially have a facultatively anaerobic lifestyle. A NiFe-Group 3a hydrogenase (COG1035) and enzymes involved in the tetrahydromethanopterin-dependent Wood–Ljungdahl pathway (COG1152, COG1880, COG1614, COG2069, COG1456) [11, 100] were detected in the common ancestor (Table S12). The synergy of abundant H_2_ in a thermal environment [101] with the Wood–Ljungdahl pathway [102] aligned with the thermal habitats of this common ancestor (Fig. 2). Principal coordinate analysis based on the orthologous gene count (Fig. S6A) indicated that the genomes of non-AOA Group-1.a/b/c clustered together, along with the Aigarchaeota. Considering the habitat of both non-AOA Group-1 and Aigarchaeota, these results suggest similarities in the microbial niches and functions between Group-1 Thaumarchaeota and Aigarchaeota [13]. The high (> 57 ºC) predicted optimal growth temperature (OGT) Group-1 Thaumarchaeota and Aigarchaeota in comparison with other Thaumarchaota groups (< 42 ºC, except two thermophic AOA (ThAOA) genomes) (Fig. S7) also support the thermophilic to mesophilic evolution of Thaumarchaota lineages.

Further timing estimation analysis revealed that the shift in OGT was well aligned with the divergence times of these groups and corresponding geological events (Fig. 6B). Specifically, Group-2 (844 Ma, 95% CI 1369–521 Ma) and Group-3.a (799 Ma, 95%CI 1417–450 Ma), which exhibit a mesophilic niche, originated around 800 Ma. This time frame coincides with the Sturtian “snowball” glaciation (717–659 Ma) [103] and the onset of large basaltic provinces (825–755 Ma)—a period termed “fire and ice” [104]. This concurrent era not only manifested extensive temperature gradients owing to the low temperature affected by the glaciation [105], but was also accompanied by intense magmatic events, which facilitated an ecological shift of extremophilic thermophiles to wide temperature niches. This proposition aligns with the results of previous research [67] and corresponds with the emergence of the initial mesophilic terrestrial group within AOA (Group I.1b—Nitrososphaerales herein: 657 Ma, 95%CI 849 – 496 Ma). This observed pattern also suggests a parallel adaptation trajectory of Thaumarchaeota from harsh environments (Group-1 Thaumarchaeota in the thermal environment) to milder habitats (Group-2 and Group-3 Thaumarchaeota in the terrestrial and ocean ecosystems).

The large gene loss combined with rare gene gain events, during the course of evolution within Group-3 Thaumarchaeota (from node 12 to node 13) (Fig. S6B) indicates genome streamlining or genome reduction phenomenon [106]. With respect to the key nodes representing the transition from non-AOA to AOA (node 12 to node 28; Fig. S6B and Table S12), the gains of genes encoding enzymes involved in ammonia oxidation and vitamin synthesis were similar to the results of comparative genomic analysis (Fig. 5) and previous studies [6, 13, 88, 107]. In addition, Group-3.a has a low proportion of arCOGs related to organic metabolism, which is similar to the pattern found in AOA groups (Fig. S7). However, other groups show equivalent numbers of organic metabolism-related arCOGs, with numbers higher than those in AOA and Group-3.a. The organic metabolism potential also supports the heterotrophy of Group-3.unk, and infers that Group-3 is the key node for the transformation from non-AOA to AOA Thaumarchaeota. Investigating more supplementary genomes of Group-3 Thaumarchaeota will expand our understanding of Thaumarcheotal evolution.

## Conclusion

Overall, this study revealed the functional contributions and phylogenetic diversity of Thaumarchaeota, the most abundant archaea in the Challenger Deep sediment of the Mariana Trench. The Thaumarchaeota in the Challenger Deep sediment may have an ecological significance considering the dominance of genes related to organic matter degradation. Global distribution analysis revealed the ubiquitous distribution of Group-3.unk and its preference for living in hadal environments. In addition, the comparative genomic analysis revealed that all binned Group-3.unk Thaumarchaeota harbors the potential for the aerobic oxidation of carbon monoxide. IPCT/DIPPS appear to be key genes for adaptation to extreme hydrostatic pressure, appearing in not only the hadal AOA as previously reported but also in non-AOA Thaumarchaeota identified in Challenger Deep sediment, while being absent in non-hadal Thaumarchaeota. Evolutionary analysis revealed the adaptation of Thaumarchaeota from thermal to moderate environments. As the closest monophyletic group to AOA, Group-3 non-AOA is a key clade for understanding the evolution of AOA.

Therefore, this study reveals a novel type of non-AOA Thaumarchaeota with a unique niche and preference for living in the hadal, thereby expanding current knowledge on Thaumarchaeota of the Challenger Deep. More cultivation and genomic information of this clade are needed to support the genomic, ecological, and evolutionary features of this new group of heterotrophic non-AOA Thaumarchaeota.

### Supplementary Information

**Additional file 1: Table S1.** Additional Genome Dataset for timing estimation. **Table S2.** Timing estimation analysis for Thaumarchaeota. **Table S3.** MW-scores for the Challenger Deep sediment metatranscriptome dataset. **Table S4.** MW-scores for the Challenger Deep sediment metagenome dataset in this study. **Table S5.** Genome description of duplicated total metagenome-assembled genomes (MAGs) in this study. **Table S6.** Genome description of Thaumarchaeota. **Table S7.** Recruited per kilobase of genome per gigabase (RPKG) values for Thaumarchaeota bins of all datasets. **Table S8.** Recruited per kilobase of genome per gigabase (RPKG) values for Thaumarchaeota bins of marine environmental datasets. **Table S9.** Transporter prediction for bins in Group-3.unk. **Table S10.** Gene annotation of five Group-3.unk bins. **Table S11.** Nitrosopumilus genomic comparative analysis for IPCT/DIPPS. **Table S12.** Gene events at key nodes (nodes 1, 12, 13, and 28) based on ancestral reconstruction.

**Additional file 2: Figure S1.** Metagenomic reads based on community constitution. Thaumarchaeota (equivalent to GTDB-Tk classification Thermoproteota) was found to be the dominant archaea in the sediment samples collected from nine different depths in the Mariana Challenger Deep. The numbers of metagenome-assembled genomes (MAGs) in each phylum are shown in brackets. MT1 to MT9 mean samples were collected from different depths: MT1, 0–2 cm; MT2, 2–3 cm; MT3, 3–4 cm; MT4, 4–5 cm; MT5, 5–6 cm; MT6, 6–7 cm; MT7; 7–8 cm; MT8, 8–9 cm; and MT9, 9–10 cm. **Figure S2.** Contig composition-independent profile of the assembled metagenome from Challenger Deep sediments. Circles represent contigs in the assembled metagenome of the MT1 sample, scaled by the square root of their length. Only contigs ≥ 5 kbp are shown. Circles are colored according to the taxonomy annotation by GTDB-Tk. **Figure S3.** Average nucleotide identity (ANI) of Group-3.unk Thaumarchaeota. MT1_thaum1 and MT7_thaum2 are metagenome-assembled genomes (MAGs) from this study. MT1_thaum1 and MT7_thaum2 have high similarity to Candidatus_Nitrosopumilus_sp_MTA1, which was binned from Mariana Trench water samples obtained at a depth of 8000 m in a previous study (1). MT1_thaum1 and other four related MAGs reconstructed from public metagenomic datasets in Group-3.unk showed high ANI value (> 0.82) with intra-group comparison but low ANI value (< 0.73) with MAGs of other groups. **Figure S4.** Occupation ratio of organic metabolism-related archaeal Clusters of Orthologous Genes (arCOGs) categories of sub-groups of Thaumarchaeota. Ammonia-oxidizing archaea (AOA) have the lowest ratio of organic metabolism COGs; however, other groups have equivalent numbers of organic metabolism-related COGs, which indicate a heterotroph habitat. Group-3.a has lower organic metabolism-related COGs than other non-AOA sub-groups and Aigarchaeota. Wilcoxon test, ^NS^ *P*>0.05, * 0.01<*P*<0.05, ** 0.001<*P*<0.01, *** *P*<0.001. **Figure S5.** Phylogenetic tree and sequence alignment of aerobic carbon monoxide dehydrogenase large subunit (CoxL) amino acid sequences. Reference sequences of Forms I and II of CoxL were selected from published papers (2) and (3-5), respectively. Nodes with bootstrap values ≥ 60 are indicated. Motif sequences of active-site configurations are indicated with asterisks. Labels in red show the MT1_thaum1 genome. All Group-3.unk members possess the CoxL. Lineages marked in purple and blue indicate Forms I and II CoxL, respectively. **Figure S6.** Orthologous gene analysis based on the evolution of Thaumarchaeota. A Principal coordinate analysis (PCoA) plot with Jaccard distance based on orthologous groups of genes in 81 selected genomes. PCoA of axes 1 vs. 2, 1 vs. 3, and 2 vs. 3 are shown. Colors represent different taxonomic groups. B Identified Clusters of Orthologous Gene (COG) functions of the gained and lost gene families of the corresponding evolutionary events. The node number is matched to the phylogenetic tree in Fig. 6. The functions of COG categories are as follows: C: energy production and conversion; D: cell cycle control, cell division, and chromosome partitioning; E: amino acid transport and metabolism; F: nucleotide transport and metabolism; G: carbohydrate transport and metabolism; H: coenzyme transport and metabolism; I: lipid transport and metabolism; J: translation, ribosomal structure, and biogenesis; K: transcription; L: replication, recombination, and repair; M: cell wall/membrane/envelope biogenesis; N: cell motility; O: post-translational modification and protein turnover; P: inorganic ion transport and metabolism; Q: secondary metabolites biosynthesis, transport, and catabolism; S: function unknown; T: signal transduction mechanisms; U: intracellular trafficking, secretion, and vesicular transport; V: defense mechanisms. **Figure S7.** Optimal growth temperature (OGT) prediction of Aigarchaeota and Thaumarchaeota groups. Aigarchaeota and Group-1 Thaumarchaeota, which are mostly found in hot springs, exhibit a high OGT. AOA and other non-AOA Thaumarchaeota have a similar relatively low OGT. **Figure S8.** Timing estimation analysis of Thaumarchaeota. The tree added 21 Euryarchaeota, 16 Crenarchaeota, one Korarchaeota, two Bathyarchaeota, and two DPANN archaea (as outgroup). Divergence nodes used for calibration are marked. Time unit at the bottom is millions of years (Ma).

## Data Availability

All binned genomes that support the findings of this study are available in the National Omics Data Encyclopedia (https://www.biosino.org) repository, OEP000774 (https://www.biosino.org/node/project/detail/OEP000744). The scripts used in this study are available at the GitHub repository: https://github.com/bikmi/NovelThaumarchaeota.

## Abbreviations

AOA: Ammonia-oxidizing archaea
Non-AOA: Non-ammonia-oxidizing archaea
DOM: Dissolved organic matter
RDOC: Refractory dissolved organic carbon
ANI: Average nucleotide identity
MAG: Metagenome-assembled genome
RPKG: Recruited per kilobase of genome per gigabase
MW-score: Metabolic weight score
ABC: ATP-binding cassette
arCOGs: Archaeal clusters of orthologous genes
VIT: Vacuolar iron transporter
CODH: Carbon monoxide dehydrogenase
IPCT/DIPPS: Inositol-1-phosphate cytidylyltransferase/di-myo-inositol-1,3′-phosphate-1′-phosphate synthase
KEGG: Kyoto Encyclopedia of Genes and Genomes
OGT: Optimal growth temperature
