# Supplementary material for: Metagenomic characterization of a novel non-ammonia-oxidizing Thaumarchaeota from hadal sediment
Source: Microbiome. 2024 Jan 8;12:7. doi: 10.1186/s40168-023-01728-2 (PMC10773090; doi:10.1186/s40168-023-01728-2)
Supplement: Supplementary file 3 — Additional file 2: Figure S1. Metagenomic reads based on community constitution. Thaumarchaeota (equivalent to GTDB-Tk classification Thermoproteota) was found to be the dominant archaea in the sediment samples collected from nine different depths in the Mariana Challenger Deep. The numbers of metagenome-assembled genomes (MAGs) in each phylum are shown in brackets. MT1 to MT9 mean samples were collected from different depths: MT1, 0–2 cm; MT2, 2–3 cm; MT3, 3–4 cm; MT4, 4–5 cm; MT5, 5–6 cm; MT6, 6–7 cm; MT7; 7–8 cm; MT8, 8–9 cm; and MT9, 9–10 cm. Figure S2. Contig composition-independent profile of the assembled metagenome from Challenger Deep sediments. Circles represent contigs in the assembled metagenome of the MT1 sample, scaled by the square root of their length. Only contigs ≥ 5 kbp are shown. Circles are colored according to the taxonomy annotation by GTDB-Tk. Figure S3. Average nucleotide identity (ANI) of Group-3.unk Thaumarchaeota. MT1_thaum1 and MT7_thaum2 are metagenome-assembled genomes (MAGs) from this study. MT1_thaum1 and MT7_thaum2 have high similarity to Candidatus_Nitrosopumilus_sp_MTA1, which was binned from Mariana Trench water samples obtained at a depth of 8000 m in a previous study (1). MT1_thaum1 and other four related MAGs reconstructed from public metagenomic datasets in Group-3.unk showed high ANI value (> 0.82) with intra-group comparison but low ANI value (< 0.73) with MAGs of other groups. Figure S4. Occupation ratio of organic metabolism-related archaeal Clusters of Orthologous Genes (arCOGs) categories of sub-groups of Thaumarchaeota. Ammonia-oxidizing archaea (AOA) have the lowest ratio of organic metabolism COGs; however, other groups have equivalent numbers of organic metabolism-related COGs, which indicate a heterotroph habitat. Group-3.a has lower organic metabolism-related COGs than other non-AOA sub-groups and Aigarchaeota. Wilcoxon test, NS P>0.05, * 0.01<P<0.05, ** 0.001<P<0.01, *** P<0.001. Figure S5. Phylogenetic tree and [file 40168_2023_1728_MOESM2_ESM.pdf]

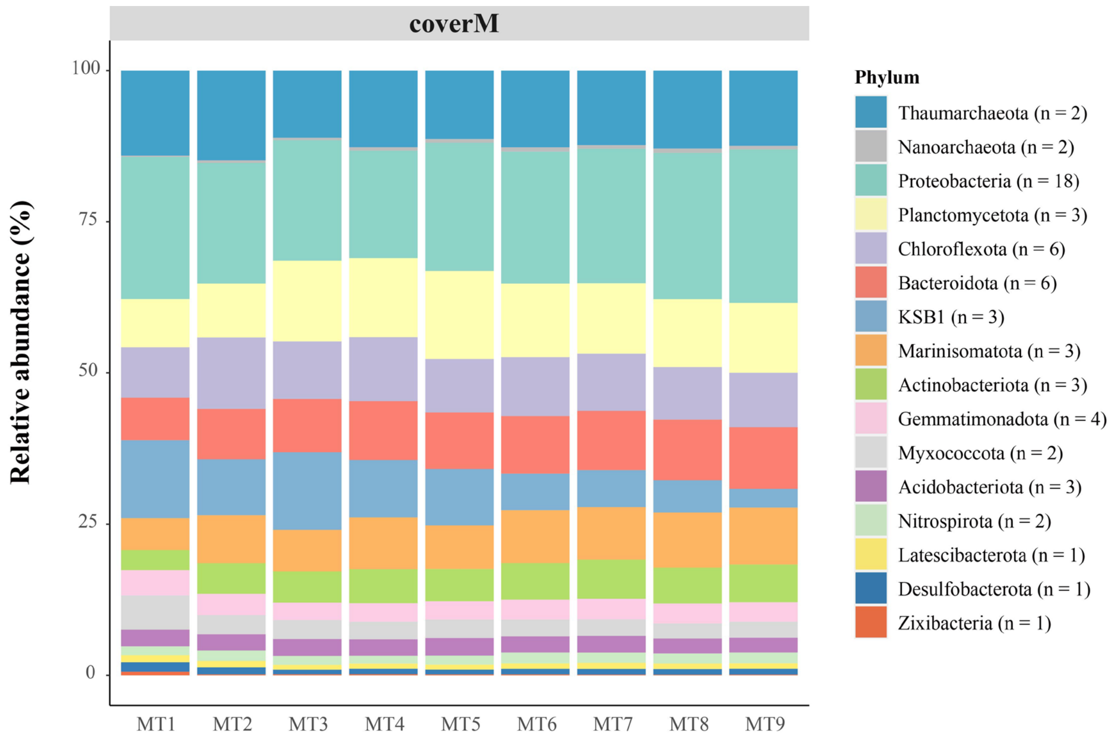

Fig. S1 Metagenomic reads based on community constitution. Thaumarchaeota (equivalent to GTDB-Tk classification Thermoproteota) was found to be the dominant archaea in the sediment samples collected from nine different depths in the Mariana Challenger Deep. The numbers of metagenome-assembled genomes (MAGs) in each phylum are shown in brackets. MT1 to MT9 mean samples were collected from different depths: MT1, 0–2 cm; MT2, 2–3 cm; MT3, 3–4 cm; MT4, 4–5 cm; MT5, 5–6 cm; MT6, 6–7 cm; MT7; 7–8 cm; MT8, 8–9 cm; and MT9, 9–10 cm.

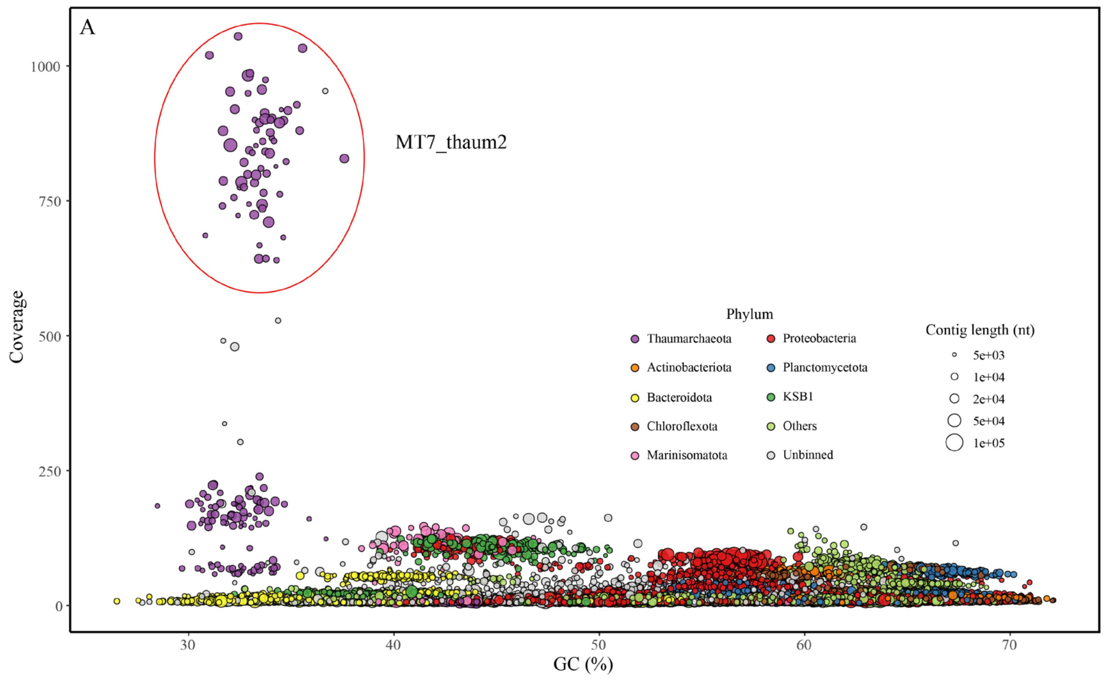

Fig. S2 Contig composition-independent profile of the assembled metagenome from Challenger Deep sediments. Circles represent contigs in the assembled metagenome of the MT1 sample, scaled by the square root of their length. Only contigs ≥ 5 kbp are shown. Circles are colored according to the taxonomy annotation by GTDB-Tk.

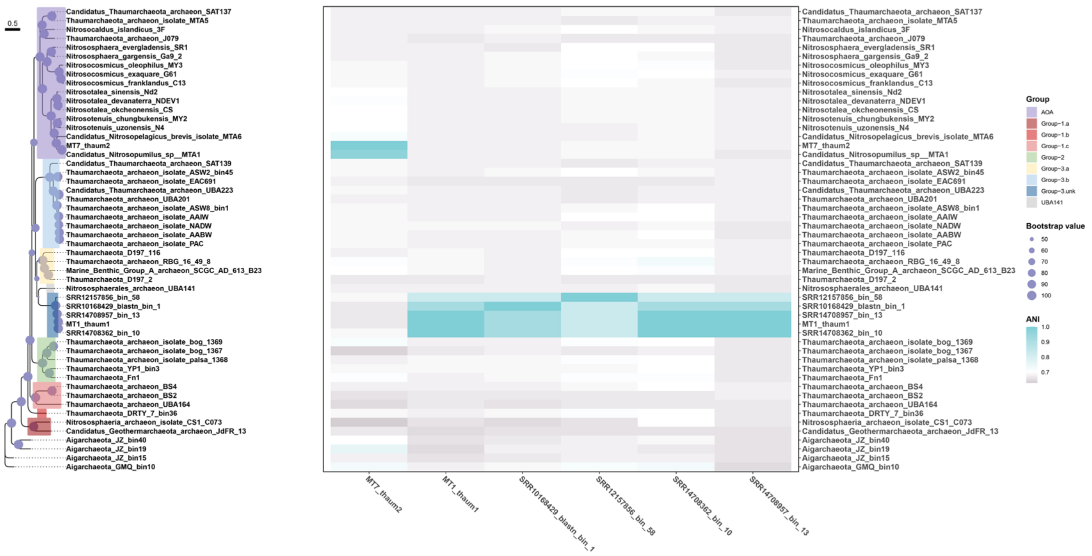

Fig. S3 Average nucleotide identity (ANI) of Group-3.unk Thaumarchaeota. MT1_thaum1 and MT7_thaum2 are metagenome-assembled genomes (MAGs) from this study. MT1_thaum1 and MT7_thaum2 have high similarity to Candidatus_Nitrosopumilus_sp_MTA1, which was binned from Mariana Trench water samples obtained at a depth of 8000 m in a previous study (1). MT1_thaum1 and other four related MAGs reconstructed from public metagenomic datasets in Group-3.unk showed high ANI value (> 0.82) with intra-group comparison but low ANI value (< 0.73) with MAGs of other groups.

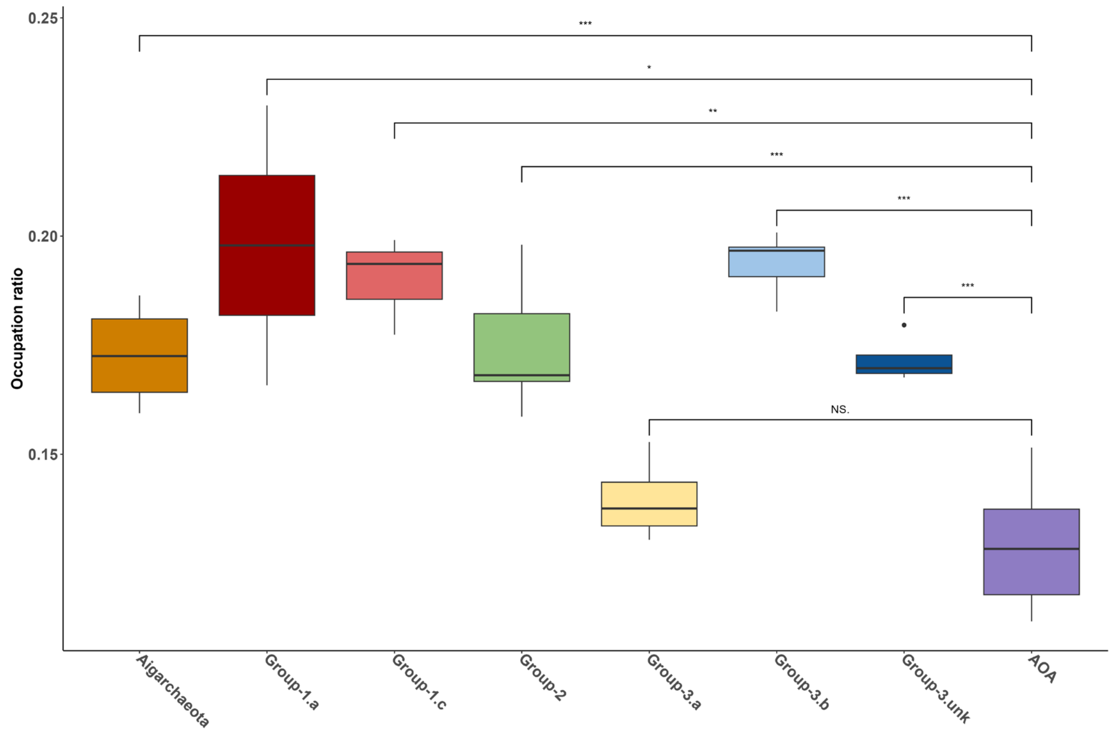

Fig. S4 Occupation ratio of organic metabolism-related archaeal Clusters of Orthologous Genes (arCOGs) categories of sub-groups of Thaumarchaeota. Ammonia-oxidizing archaea (AOA) have the lowest ratio of organic metabolism COGs; however, other groups have equivalent numbers of organic metabolism-related COGs, which indicate a heterotroph habitat. Group-3.a has lower organic metabolism-related COGs than other non-AOA sub-groups and Aigarchaeota. Wilcoxon test, ^{NS} $P > 0.05$, * $0.01 < P < 0.05$, ** $0.001 < P < 0.01$, *** $P < 0.001$.

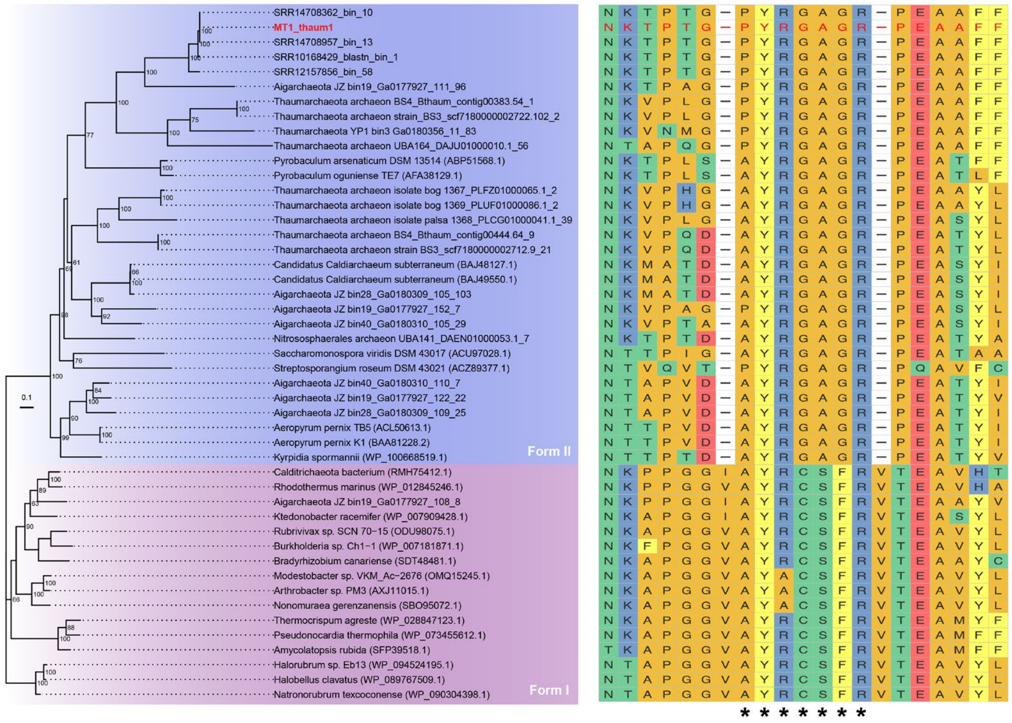

Fig. S5 Phylogenetic tree and sequence alignment of aerobic carbon monoxide dehydrogenase large subunit (CoxL) amino acid sequences. Reference sequences of Forms I and II of CoxL were selected from published papers (2) and (3-5), respectively. Nodes with bootstrap values ≥ 60 are indicated. Motif sequences of active-site configurations are indicated with asterisks. Labels in red show the MT1_thaum1 genome. All Group-3.unk members possess the CoxL. Lineages marked in purple and blue indicate Forms I and II CoxL, respectively.

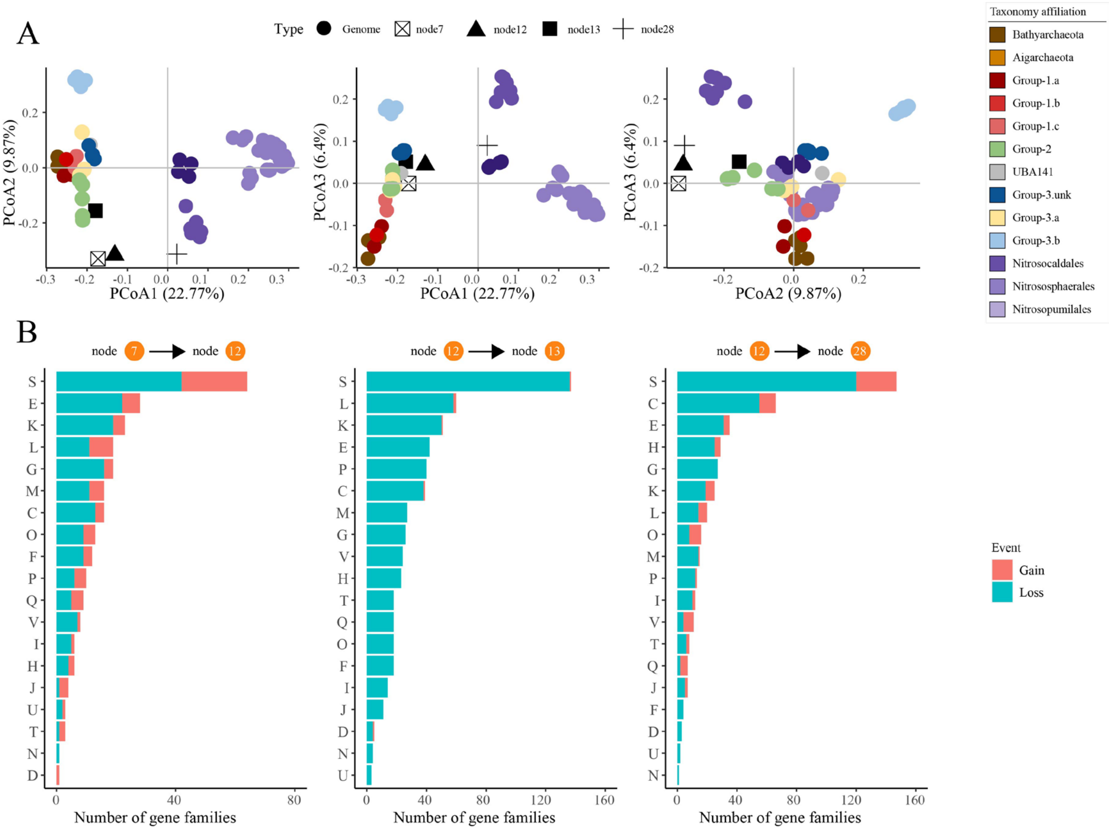

Fig. S6 Orthologous gene analysis based on the evolution of Thaumarchaeota. A Principal coordinate analysis (PCoA) plot with Jaccard distance based on orthologous groups of genes in 81 selected genomes. PCoA of axes 1 vs. 2, 1 vs. 3, and 2 vs. 3 are shown. Colors represent different taxonomic groups. **B** Identified Clusters of Orthologous Gene (COG) functions of the gained and lost gene families of the corresponding evolutionary events. The node number is matched to the phylogenetic tree in Fig. 6. The functions of COG categories are as follows: C: energy production and conversion; D: cell cycle control, cell division, and chromosome partitioning; E: amino acid transport and metabolism; F: nucleotide transport and metabolism; G: carbohydrate transport and metabolism; H: coenzyme transport and metabolism; I: lipid transport and metabolism; J: translation, ribosomal structure, and biogenesis; K: transcription; L: replication, recombination, and repair; M: cell wall/membrane/envelope biogenesis; N: cell motility; O: post-translational modification and protein turnover; P: inorganic ion transport and metabolism; Q: secondary metabolites biosynthesis, transport, and catabolism; S: function unknown; T: signal transduction mechanisms; U: intracellular trafficking, secretion, and vesicular transport; V: defense mechanisms.

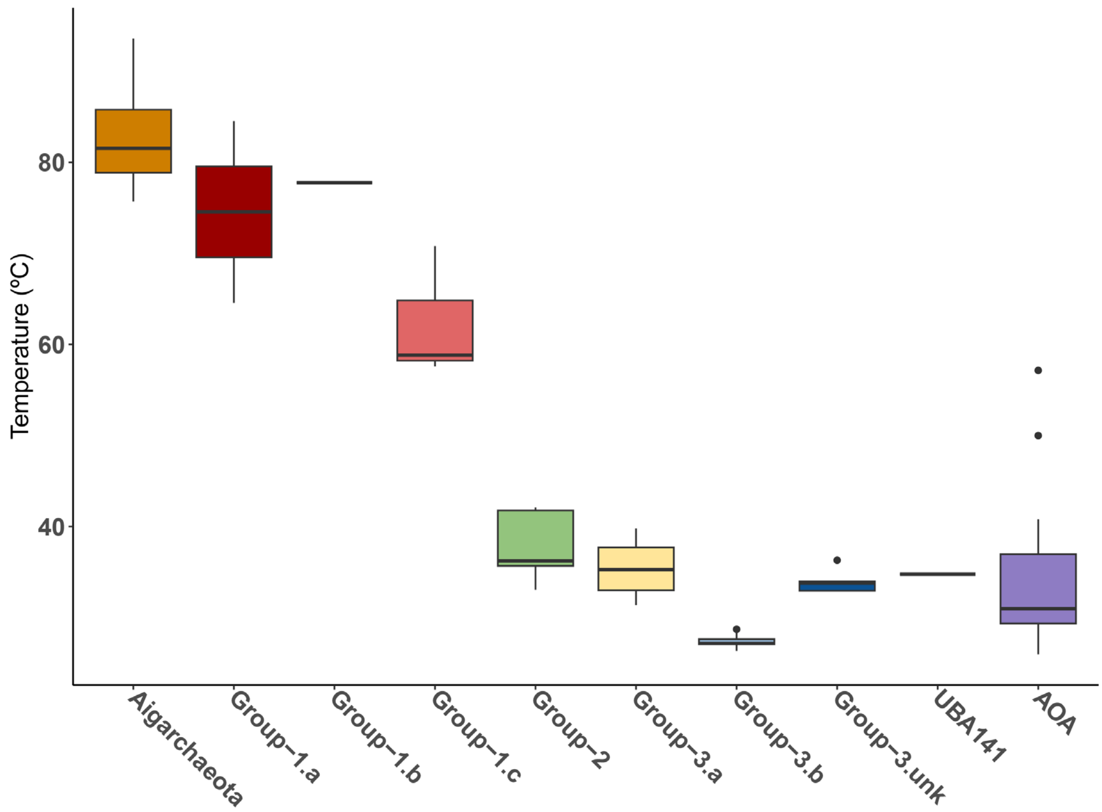

Fig. S7 Optimal growth temperature (OGT) prediction of Aigarchaeota and Thaumarchaeota groups. Aigarchaeota and Group-1 Thaumarchaeota, which are mostly found in hot springs, exhibit a high OGT. AOA and other non-AOA Thaumarchaeota have a similar relatively low OGT.

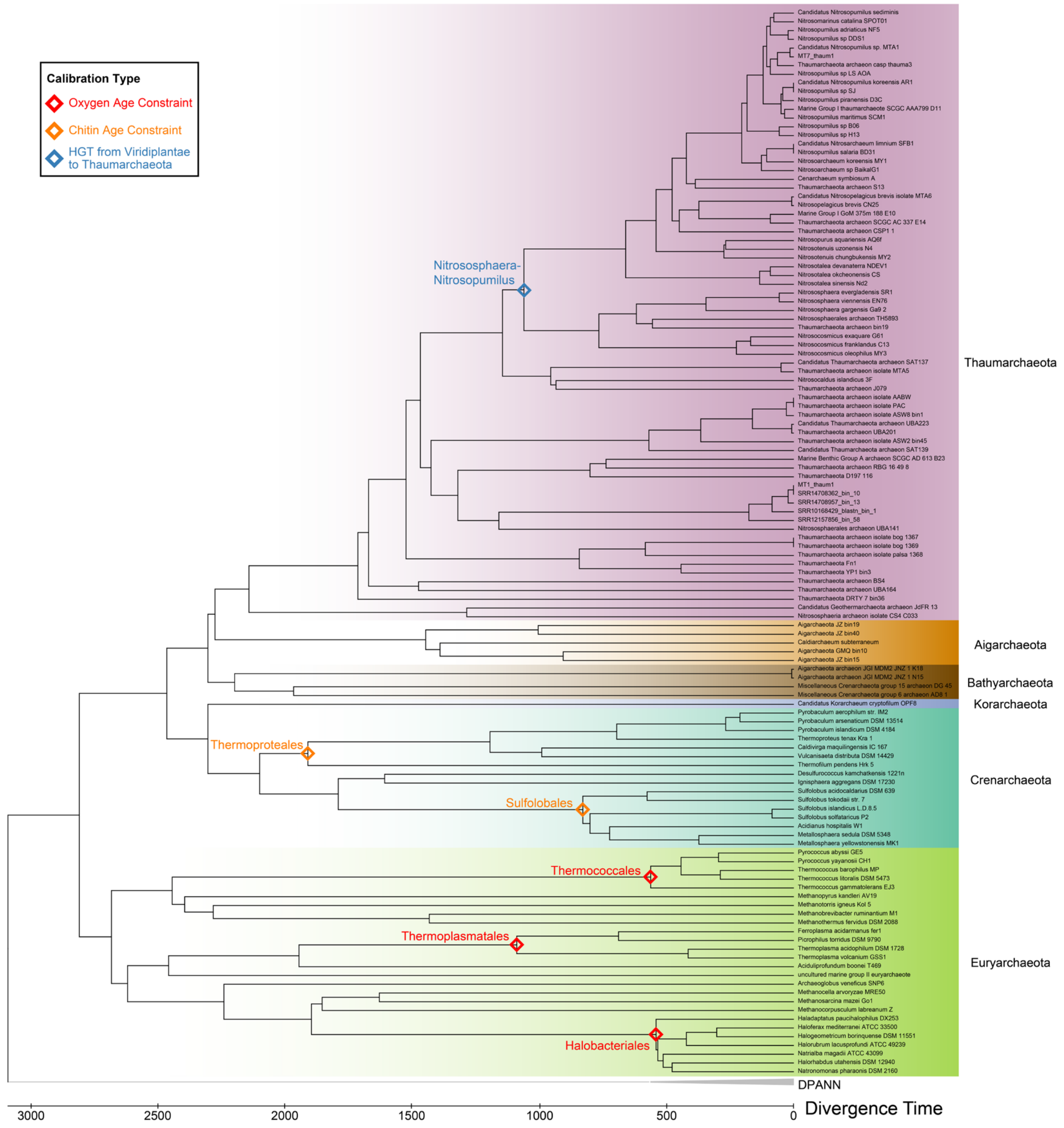

Fig. S8 Timing estimation analysis of Thaumarchaeota. The tree added 21 Euryarchaeota, 16 Crenarchaeota, one Korarchaeota, two Bathyarchaeota, and two DPANN archaea (as outgroup). Divergence nodes used for calibration are marked. Time unit at the bottom is millions of years (Ma).

References

1. Zhong H, Lehtovirta-Morley L, Liu J, Zheng Y, Lin H, Song D, et al. Novel insights into the Thaumarchaeota in the deepest oceans: their metabolism and potential adaptation mechanisms. Microbiome. 2020; 8(1):78.
2. Cordero PRF, Bayly K, Man Leung P, Huang C, Islam ZF, Schittenhelm RB, et al. Atmospheric carbon monoxide oxidation is a widespread mechanism supporting microbial survival. ISME J. 2019; 13(11):2868-2881.
3. Hua ZS, Qu YN, Zhu Q, Zhou EM, Qi YL, Yin YR, et al. Genomic inference of the metabolism and evolution of the archaeal phylum Aigarchaeota. Nat Commun. 2018; 9(1):2832.
4. Hogendoorn C, Pol A, Picone N, Cremers G, van Alen TA, Gagliano AL, et al. Hydrogen and carbon monoxide-utilizing *Kyrpidia spormannii* species from Pantelleria Island, Italy. Front Microbiol. 2020;

11:951.
5. Quiza L, Lalonde I, Guertin C, Constant P. Land-use influences the distribution and activity of high affinity CO-oxidizing bacteria associated to type I-*coxL* genotype in soil. Front Microbiol. 2014; 5:271.
